# Using a Gun Camera

**Published:** 1882-06

**Authors:** 


### USING A GUN CAMERA.

In taking instantaneous photographs it is well known that some difficulty is experienced in bringing the object into the field of the camera. The process of taking aim at, for instance, a moving object, such as a ship, has sometimes to be repeated several times, and in the end result is unsatisfactory, M. Marrey has, to get over this difficulty, designed a photographic gun. This is neither more nor less than a very large revolver, with a stock to put to the shoulder. The barrel is a telescope—that is to say, it contains the lenses of a camera ; there are sixteen apertures, which take the place of the chambers. The photographer puts in a sensitized plate behind these apertures, and performing an operation analogous to cocking the weapon, he is ready for the field.

On seeing a flying bird he takes aim and pulls the trigger, the chamber revolves once, and in one second he obtains sixteen little pictures of the bird in various positions. Hitherto M. Marrey has made the use of his photographic gun for the purpose of investigating the flight of birds. In this case clearness of definition is of little consequence, so long as a dark image or silhouette the shape of the bird as obtained, so that it matters little whether the object aimed at be focused or not ; but it is obvious that in a multitude of other cases the image can be obtained perfectly in focus. Indeed, it will be seen that the system of thus carrying a small camera to be steadied against the shoulder admits of the utmost service to the photographer.

